# Toxoplasmic encephalitis after allogeneic hematopoietic stem cell transplantation

**DOI:** 10.14744/nci.2020.66049

**Published:** 2022-05-11

**Authors:** Feyza Izci Cetinkaya, Necati Mumcu, Gamze Kalin Unuvar, Aysegul Ulu Kilic, Leylagul Kaynar

**Affiliations:** 1 Department of Infectious Diseases, Erciyes University Faculty of Medicine, Kayseri, Turkiye; 2 Department of Hematology, Erciyes University Faculty of Medicine, Kayseri, Turkiye

**Keywords:** Allogeneic stem cell transplantation, encephalitis, toxoplasmosis

## Abstract

Cerebral toxoplasmosis is a rare but often life-threatening infection after hematopoietic stem cell transplantation (HSCT). In such cases, early diagnosis and initiation of treatment are vital. We describe a case of cerebral toxoplasmosis in a patient who underwent HSCT for acute myeloid leukemia. Infection was diagnosed with polymerase chain reaction (PCR) test of cerebrospinal fluid and cranial magnetic resonance imaging scan. The patient treated with trimethoprim-sulfamethoxazole and clindamycin combination. However, she died from nosocomial infection after 15 days of treatment. This life-threatening infection should be considered in a patient who is post-HSCT present with neurologic symptoms and brain lesions. PCR is an important and rapid diagnostic tool for toxoplasmosis. Cranial imaging scan and PCR should be used together to diagnosed.

Toxoplasmosis is caused by an intracellular protozoan parasite *Toxoplasma gondii* and mostly associated with a self-limiting disease in an immunocompetent host. Infection is mainly transmitted by ingestion of food or water contaminated with oocysts. Furthermore, the disease may be acquired by eating undercooked or raw meat of animals containing tissue cysts [1]. In immunocompromised patients, disease may occur as a severe infection due to the reactivation of latent cysts but also sometimes may be acquired as a primary infection [2].

This infection in allogeneic hematopoietic stem cell transplantation (HSCT) recipients most often occurs in the first 6-month post-transplantation (the majority occur in the first 30–90 days). The incidence of toxoplasmosis after HSCT has been reported to vary between 0.4% and 8.7%, depending on the endemic regions [3]. Especially, in patients underwent HSCT, it is uncommon but life-threatening infection and with a high mortality rate (60–90%) [4]. The clinical manifestations are such as encephalitis, pneumonia, and disseminated infections in HSCT patients [2]. In a study, 27 of 655 HSCT recipients had developed central nervous system infection after transplantation, of which 20 (74%) were considered as toxoplasma encephalitis (TE) [5]. Patients with TE typically present with headache, confusion, fever, drowsiness, hemiparesis, reflex change, and convulsions [1]. Diagnosis is usually based on clinical features, evidence of serum antibodies (IgM, IgG), and done by real-time polymerase chain reaction (PCR) (on blood or cerebrospinal fluid [CSF]). Studies have reported 50–65% sensitivity and 95–100% specificity for PCR techniques for the detection of parasites. Although PCR is not routinely used, it is a non-invasive important test for early diagnosis of seropositive patients. Cranial magnetic resonance imaging (MRI) can use for diagnosis, but these findings are non specific. Therefore, difficulties have been associated with the antemortem diagnosis of toxoplasmosis post-transplant patients [6].

We report a case with cerebral toxoplasmosis after following HSCT that was diagnosed with using PCR techniques and brain MRI methods.

## CASE REPORT

**Figure 1:**
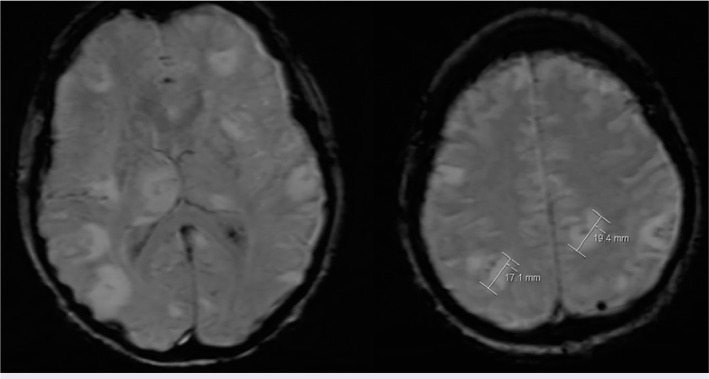
Multiple lesions are hyperintense on T2-weighted MRI with focal noduler after administration of contrast.

A 53-years-old female patient with acute myeloid leukemia underwent HSCT. She received allogeneic peripheral stem cell transplant from her human leukocyte antigen matched sister with TEC-RIC protocol (thiotepa, cyclophosphamide, etoposide, busulfan, fludarabine, cyclosporine, mycophenolate mofetil). Before allogenic SCT, she received intravenous meropenem and vancomycin for a neutropenic fever episode and colitis. Furthermore, intravenous 20 mg/day methylprednisolone for 15 days was initiated due to a possible engraftment fever. Forty-two days later after the transplantation, she developed a new episode of fever with new-onset headache and confusion. Furthermore, the patient had stiff neck. Physical examination findings were unclear because of the patient’s confusion. MRI scan of the brain showed lesions in both thalamus and basal ganglia levels with multiple peripheral contrast which increases after injection of intravenous contrast agent (Fig. 1). Cranial MRI showed cerebritis which is thought to be associated with opportunistic infections or leukemic infiltration. Hence, her treatment was changed; meropenem dose doubled (6 g/day) and ampicillin (8 g/day) added for suspected opportunistic infections. Furthermore, methylprednisolone was stopped and instead dexamethasone was added treatment for elevated intracranial pressure.

The patient thereafter exhibited progressive neurological disturbances, so she was transferred to the intensive care unit and was intubated. Lumbar puncture was remarkable for a high protein level of 375 mg/dl (normal 14–45) but also with normal levels of CSF glucose and white blood cell count. Cytomegalovirus PCR, herpes PCR (type 1/2), mycobacterium PCR were negative in CSF; also, no growth was noted in fungal culture. CSF toxoplasma DNA was detected positive with PCR. The patient was known to be seronegative as toxoplasmosis IgM and IgG antibodies were detected negative before transplantation. To confirm the diagnosis of toxoplasmosis, invasive procedures such as biopsy were avoided because the patient had thrombocytopenia. However, TE was considered with the current findings and intravenous trimethoprim-sulfamethoxazole (TMP-SMX) (TMP dose was 10 mg/kg), clindamycin 600 mg I.V. (3 times a day) combination therapy was started. In spite of the therapy, the patient was followed with a Glasgow Coma Scale score of 3 and required inotropic support with noradrenaline. She died due to ventilator-associated pneumoniae after 15 days of treatment and the control of MRI could not be obtained.

## DISCUSSION

Toxoplasmosis is an opportunistic infectious disease in immunocompromised patients. Furthermore, TE is a life-threatening infection in these patients; thus, early diagnosis and initiating appropriate treatment are essential to improve survival [3]. In spite of the low incidence of in our (<0.5%), the mortality rate of toxoplasmosis was reported 60–90% in the United States [4]. Patients have a complicated medical history with immunosuppression, especially after HSCT. Diagnosis of toxoplasmosis in HSCT receipts requires strong clinical suspicion because the clinical symptoms of the infection are often masked by immunosuppressive therapy [7]. Therefore, TE must be carefully considered in the differential diagnosis of a wide spectrum of clinical presentations. Speech abnormality and hemiparesis are the most common initial focal findings of this infection. In this presented neutropenic patient, headache and confusion were the first developed symptoms [6, 8]. The patient who had clinical signs of the involvement of central nervous system was evaluated for potential clinical problems such as leukemic encephalopathy, tuberculosis, toxoplasmosis, and meningoencephalitis. However, since the patient was followed up in the clinic, she was diagnosed with clinical findings before the associated symptoms developed in the early period [9].

Cranial MRI is an important diagnostic method in the diagnosis of brain lesions. Patients who are immunocompromised are susceptible to a variety of opportunistic infections and malignancies, identifying a single cause that is responsible for the patient’s symptoms is often difficult with imaging findings. Toxoplasmosis may occur as a brain mass lesion most commonly in immunosuppressive patients (AIDS etc.), whereas malignant lymphoma commonly may manifest as a brain neoplastic lesion. MR findings of TE are similar to other brain lesions such as brain tumor and abscess. Therefore, the other differential diagnosis methods of TE are necessary [10, 11]. The early biopsy is recommended for confirmation of diagnosis. These techniques are used less frequently because of the difficulty of obtaining these specimens [4]. In this case, although MRI findings indicated TE, it may also be leukemic infiltration or other infectious pathogens. MRI showed lesions with multiple peripheral contrast increases after intravenous contrast agents injection at both thalamus and basal ganglia levels [8, 10]. Brain biopsy was considered to be a final option in our case. As the patient has thrombocytopenia, brain tissue sample could not be taken for the risk of bleeding. In addition, the location of the lesions was not appropriate for biopsy.

TE is generally diagnosed using a toxoplasma antibody titer. Therefore, TE cannot be ruled out in for immunosuppressed patients, even if serum anti-toxoplasma antibody test is negative [4].

PCR technique which is convenience and rapid, has been usually used for the diagnosis of cerebral toxoplasmosis. When clinical symptoms and signs indicate an involvement of the central nervous system of toxoplasmosis, cranial imaging should be performed and obtained CSF after a lumbal puncture for Toxoplasma DNA detection by PCR [12]. PCR for the diagnosis of TE is the possibility of obtaining false-negative results. Sensitivity and specificity were reported at 33–83% and 96–100%, respectively [13, 14]. Cibickova et al. [15] studied patients after HSCT and stated that negative results of CSF PCR should not rule out the presence of disease. Therefore, a positive result on PCR indicates a diagnosis of TE, but a negative result on PCR should not rule out the presence of disease. In immunosuppressive patients, medical treatment and prophylaxis of disease should be the first steps because misdiagnosis and delayed treatment could be life-threatening. TMP/SMX prophylaxis is used routinely for Pneumocystis jirovecii infection. Furthermore, this prophylaxis provides protection against reactivation of toxoplasmosis. Occasionally, breakthrough infections can occur during prophylaxis as in our case. The standard treatment of toxoplasmosis is a combination of pyrimethamine and sulfadiazine, but these drugs are not available in Turkey. A combination of TMP-SMX and clindamycin is used as an alternative treatment as we also initiated. The study of Hakko et al. showed that combination of TMP/SMX and clindamycin found to be associated with improvement of cerebral toxoplasmosis three of five patients [3]. Unfortunately, the presented case died associated with nosocomial infection, that’s why the response of treatment could not be assessed.

## Conclusion

In conclusion, this uncommon but life-threatening infection should be considered in a patient who post-HSCT presents with neurologic symptoms and brain lesions. PCR is an important and rapid diagnostic tool for TE. PCR and cranial imaging methods should be used together to diagnose, as an alternative of more invasive procedures such as brain biopsy. In this way, survival can be increased with early diagnosis and treatment.
